# Molecular Alterations in Paired Epithelial Ovarian Tumors in Patients Treated with Neoadjuvant Chemotherapy

**DOI:** 10.3390/cancers16213580

**Published:** 2024-10-24

**Authors:** Adamantia Nikolaidi, Eirini Papadopoulou, Dimitrios Haidopoulos, Michalis Liontos, Elena Fountzilas, Georgios Tsaousis, Kalliroi Goula, Eleftheria Tsolaki, Athina Christopoulou, Ioannis Binas, Sofia Stamatopoulou, Anna Koumarianou, Sofia Karageorgopoulou, Anna Goussia, Amanda Psyrri, Christos Papadimitriou, Helen Gogas

**Affiliations:** 1 Oncology Clinic, Mitera Hospital, 15123 Athens, Greece; 2 Genekor Medical SA, 15344 Athens, Greece; eirinipapad@genekor.com (E.P.); gtsaousis@genekor.com (G.T.); 3 First Department of Obstetrics and Gynaecology, Division of Gynecologic Oncology, Alexandra Hospital, National and Kapodistrian University of Athens, 11528 Athens, Greece; dimitrioshaidopoulos@gmail.com; 4 Department of Clinical Therapeutics, Alexandra General Hospital, School of Medicine, National and Kapodistrian University of Athens, 11528 Athens, Greece; mliontos@gmail.com; 5 Department of Medical Oncology, St Luke’s Clinic, 55236 Thessaloniki, Greece; fountzila@oncogenome.gr; 6 German Oncology Center, European University Cyprus, 1516 Nicosia, Cyprus; 7 Department of Pathology, Alexandra Hospital, 11528 Athens, Greece; kallirroigoula8@gmail.com; 8 Laboratory of Molecular Oncology, Hellenic Foundation for Cancer Research/Aristotle University of Thessaloniki, 54006 Thessaloniki, Greece; e_tsolaki@hecog.ondsl.gr; 9 Medical Oncology Unit, S. Andrew Hospital, 26332 Patras, Greece; athinachristo@hotmail.com; 10 Second Department of Medical Oncology, Metropolitan Hospital, 18547 Piraeus, Greece; ioannisbinas@gmail.com; 11 Oncology Unit, General Hospital of Kalamata, 24100 Kalamata, Greece; stamatopouloudoubali@yahoo.gr; 12 Hematology-Oncology Unit, Fourth Department of Internal Medicine, Attikon University Hospital, Medical School, National and Kapodistrian University of Athens, 12462 Athens, Greece; akoumari@yahoo.com; 13 Third Department of Medical Oncology, IASO Clinic, 15123 Athens, Greece; skarageorgopoulou@iaso.gr; 14 Department of Pathology, University Hospital of Ioannina, 45500 Ioannina, Greece; agoussia@uoi.gr; 15 Section of Medical Oncology, Department of Internal Medicine, Attikon University Hospital, Faculty of Medicine, National and Kapodistrian University of Athens, 12462 Athens, Greece; dpsyrri@med.uoa.gr; 16 Oncology Unit, Aretaieion University Hospital, National and Kapodistrian University of Athens, 11528 Athens, Greece; cpapadim@med.uoa.gr; 17 First Department of Medicine, National and Kapodistrian University of Athens, 11527 Athens, Greece; hgogas@med.uoa.gr

**Keywords:** neoadjuvant chemotherapy (NACT), genomic alterations, genomic instability score (GIS), tumor cell content (TCC), next-generation sequencing (NGS), chemotherapy response score (CRS)

## Abstract

Background: Neoadjuvant chemotherapy (NACT) followed by interval debulking surgery (IDS) and adjuvant chemotherapy is a therapeutic choice for women with advanced ovarian cancer. Whether NACT affects the tumor’s molecular profile has not been determined. Methods: This was a retrospective study of patients with advanced-stage epithelial ovarian cancer treated with NACT at oncology departments affiliated with the Hellenic Cooperative Oncology Group (HeCOG). Tumor molecular profiling was performed on formalin-fixed and paraffin-embedded (FFPE) tumor pre- and post-NACT tissues. Homologous recombination deficiency (HRD), tumor-infiltrating lymphocytes (TILs), tumor molecular alterations, and tumor mutational burden (TMB) via next-generation sequencing analysis were assessed. Results: Overall, tumors from 36 patients were assessed, and molecular profiling was evaluated in 20 paired tumor samples. HRD positivity exhibited no significant change between pre- and post-NACT tumors. The BRCA1/2 mutational status remained constant, irrespective of the treatment administration. Pre-NACT tumors tended to exhibit a lower percentage of intratumoral TILs compared to post-NACT tumors (*p* = 0.004). Differences in the mutation profile between pre- and post-treatment tissue were observed in 33.33% (6/18) of the cases. The mean tumor cell content (TCC) (*p*-value: 0.0840) and the mean genomic instability score (*p*-value: 0.0636) decreased slightly numerically after therapy. A moderate inverse relationship was observed between the pre-NACT TMB and the chemotherapy response score (*p*-value: 0.038), indicating this correlation is statistically significant. Conclusion: This study provides insights into the effect of NACT on the tumor molecular landscape. While BRCA1/2 and HRD status remained stable, an increase in TIL proportion and changes in the mutational profiles were observed post-treatment.

## Simple Summary

The standard of care for most women with advanced ovarian cancer is primary cytoreduction followed by platinum-based chemotherapy and paclitaxel. However, the use of neoadjuvant chemotherapy (NACT) followed by interval debulking surgery (IDS) and adjuvant chemotherapy is not inferior, according to prospective randomized trials. Ovarian cancer has a low mutational load but exhibits a high detection rate of molecular alterations in various genes. It remains unclear whether this is an intrinsic feature of ovarian cancer or if it is due to the administration of a platinum combination during NACT. Our study aimed to determine whether NACT affects the tumor’s molecular profile. Any modifications in these areas would need to be identified in the initial therapeutic planning, especially in the era of poly (ADP-ribose) polymerase (PARP) inhibitors.

## 1. Introduction

The ovarian, fallopian tube, and primary peritoneal high-grade cancer (OVCA) is the seventh leading cause of cancer death in women in the US, accounting for more deaths than any other cancer of the female reproductive system. The standard of care for most of these women has been primary cytoreduction (PCS) followed by platinum-based chemotherapy and paclitaxel [1]. However, prospective randomized trials have evaluated the use of neoadjuvant chemotherapy (NACT) followed by interval debulking surgery (IDS) and adjuvant chemotherapy [2,3,4,5,6,7,8,9].

Recently, due to the high incidence of deficient DNA repair through homologous recombination in ovarian cancer, PARP inhibitors have emerged as an important therapeutic option. According to the latest guidelines, PARP inhibitors are indicated as a maintenance treatment for adult patients with advanced International Federation of Gynecology and Obstetrics (FIGO) stage III or IV cancers. Olaparib is recommended as a monotherapy for patients with the BRCA1/2 mutation [9], in combination with bevacizumab for patients with homologous recombination deficiency (HRD) [10] or niraparib for all patients [11]. Ovarian cancer has a low mutational load but exhibits a high detection rate of molecular alterations in various genes. To date, it remains unclear whether this is an intrinsic feature of ovarian cancer or if it is due to the administration of a platinum combination during NACT. Moreover, a well-documented phenomenon in patients with ovarian cancer who carry inherited pathogenic genetic variants is the somatic reversal of these genetic lesions following platinum treatment [12]. This reversal significantly contributes to the resistance to PARP inhibitors [13]. This same phenomenon was described in a series of patients whose tumors were analyzed at different time points during treatment with a platinum regimen and olaparib [14]. Furthermore, NACT may enhance the host immune response by significantly affecting the tumor microenvironment (TME) through the increase of the CD8+ and T helper cell populations [15,16,17].

To address these open questions and understand the effect of neoadjuvant chemotherapy (NACT) on tumor biology, we examined paired tumor samples from patients with advanced ovarian cancer who received NACT. Our study aimed to determine whether preoperative chemotherapy affects the tumor’s molecular profile, including HRD status, gene mutations, and tumor mutational burden (TMB).

## 2. Patients and Methods

Τhis study included patients over 18 years old with epithelial ovarian cancer stage FIGO III or IV, treated with NACT at oncology departments affiliated with the Hellenic Cooperative Oncology Group (HeCOG). Treatment was administered per physician’s assessment and based on international guidelines. Patients were identified from a specially designed HECOG database, and all clinical data were recorded from this database. All living patients who participated in the study provided written informed consent for the use of their biological material for research purposes. A waiver of consent was obtained for the use of biological material and pseudo-anonymized data from deceased patients. The study was conducted from 2018 to 2024, in accordance with the Declaration of Helsinki, and was approved by the Ethics Committee of the Aristotle University of Thessaloniki (protocol code 403, 9 February 2018) for studies involving humans. In total, 72 paired FFPE tissue samples were collected from 36 patients with ovarian cancer before and after NACT. After the TCC assessment, three patients were excluded from the study due to insufficient pretreatment of tumor tissue. In addition, ten patients were omitted from the analysis due to insufficient TCC in the tissue sample acquired after NACT.

### 2.1. Tumor Infiltrating Lymphocytes (TIL) Assessment

TILs were evaluated on hematoxylin-eosin-stained slides, according to the recommendations published by the TILs Working Group, in both intratumoral areas (iTILs) as well as in tumor stroma (sTILs) [18].

### 2.2. DNA Extraction

The genomic DNA isolation from formalin-fixed and paraffin-embedded (FFPE) tumor biopsies was carried out on tumor areas with a tumor cell content (TCC) of over 25%, which measured at least 3 mm × 3 mm. This was done using the MagMAX™ Total Nucleic Acid Isolation Kit from Thermo Fischer Scientific, Waltham, MA, USA. The procedures were performed according to the instructions provided by qualified pathologists on sections stained with hematoxylin and eosin. Samples were excluded from molecular analysis if they exhibited a tumor cell content below 25%. In addition, samples with insufficient tumor size and a total quantity of 200 ng in isolated DNA were also excluded since they were not compatible with the subsequent test requirements.

### 2.3. NGS Procedure

Targeted next-generation sequencing (NGS) analysis was performed using the Oncology Multi-Gene Variant Assay (GenePlus, Beijing, China), a qualitative in vitro diagnostic test that detects variants in 1021 tumor-related genes and gene rearrangements/fusions in 38 genes. Sequencing was carried out on an MGI sequencing platform (DNBSEQ-G400). The analysis included the entire exon regions of 312 genes, introns/promoters/fusion breakpoint regions of 38 genes, and partial coding exons of 709 genes (Supplementary Table S1). The test also reported on 30+ immune response biomarkers, including the tumor mutation burden (TMB) score.

### 2.4. Data Analysis and Result Interpretation

The sequencing data were analyzed using the supporting bioinformatics analysis and result interpretation instrument Gene+ Box to obtain the gene variation information of the sample. SNV/Indel/SV are identified as positive in the case of a mutation frequency ≥1% for FFPE samples and ≥1% for plasma samples.

### 2.5. OncoScan CNV Assay

The OncoScan™ CNV Assay (Thermo Fisher Scientific, Waltham, MA, USA) was carried out as previously described [19]. The Chromosome Analysis Suite (ChAS) was used for the primary analysis of the CEL files and quality control calculations (MAPD, ndSNPQC). ASCAT (v3.0.0) (allele-specific copy number analysis of tumors), using logR ratio and B-allele frequency of autosomal markers with GC content and replication timing correction, was used to evaluate and calculate tumor purity, ploidy, and allele-specific copy number profiles.

### 2.6. Statistical Analysis

The *p*-values were calculated utilizing Fisher’s exact test. Data visualizations were generated using the seaborn and matplotlib libraries in Python v3.8.16, and the Wilcoxon signed-rank test was conducted to determine the statistical differences in VAF values before and after therapy. A *p*-value < 0.05 was considered to be statistically significant. The Plotly.js charting library was used to generate box plots. Spearman’s rank correlation coefficient (rho) was calculated.

## 3. Results

### 3.1. Patients

Overall, 36 patients with advanced disease were deemed unresectable at presentation and received NACT to achieve cytoreduction. In the majority of patients (88.8%), the diagnosis was established by biopsy, while four patients had cytological confirmation (cell block) of HGSOC. Approximately half of the patients (52.7%, or 19/36) presented with stage III HGSOC. Additionally, 41.6% of patients (15/36) achieved a Complete Response Score (CRS1), whereas 33.3% (12/36) achieved CRS2 and 25.1% (9/36) achieved CRS3. There was no disease progression during NACT, and all patients underwent IDS. Patient characteristics are shown in Table 1.

### 3.2. Tumor Tissue Samples

Sufficient TCC (TCC > 25%) was detected in 27 tissue samples before treatment and in 25 samples following treatment. Furthermore, the GIS analysis could not be completed for three paired samples due to the inadequate quality of the genetic material. Overall, 40 FFPE samples from 20 patients proceeded for further evaluation (Supplementary Figure S1, Consort diagram).

### 3.3. Pre- and Post-NACT TIL Evaluation

Pre-NACT tumors tended to exhibit a lower percentage of TILs (median value: sTILs 20%, and iTILs 1%) compared to post-NACT tumors (median value: sTILs 40%, and iTILs 2%), with the iTILs reaching statistical significance (*p* = 0.00411) but not the sTILs (*p* = 0.0857). However, there was no discernible association between CRS and either post or pre-TIL expression.

### 3.4. Pre- and Post-NACT HRD Analysis

Among the 20 tissue samples from the treatment-naïve cases evaluated, HRD was observed in 40% (8/20) of the cases, including seven with elevated GIS > 42 and one with a low GIS but with a BRCA1 alteration present. An examination of the patients following NACT identified 35% (7/20) as HRD-positive. In two patients, the GIS status differed between the paired samples: in one of these patients, the GIS status detected became negative in the second tumor tissue sample, whereas in the second patient, it became positive. Thus, a patient who was previously HRD-negative could be reclassified as HRD-positive after NACT, and vice versa. Overall, among 20 evaluable pairs (N = 40 samples), a moderate intrapatient correlation in the HRD score values was observed (r^2^ = 0.369). A BRCA1/2 mutation was identified in 20% (4/20) of the patients; however, the BRCA1/2 mutational status remained constant irrespective of the treatment administration.

### 3.5. Pre- and Post-NACT Comprehensive Genomic Profile (CGP)

In addition, a CGP analysis was performed on all 40 samples with evaluable HRD results. Successful analysis for both pre- and post-treatment samples was achieved in 18 patients. Sixty-two variants were detected pre-NACT and fifty-three post-NACT (Supplementary Table S2). Differences in the mutation profile between pre- and post-treatment tissue were observed in 33.33% (6/18) of the cases, indicating a significant impact of the treatment on tumor biology or significant tumor heterogeneity.

The most prevalent alterations were found in the key role player TP53 gene, as evidenced by the detection of 57.89% in the pre-treatment samples, as per previous reports [20]. Post-treatment tissue analysis revealed the absence of the TP53 mutation in one patient, whereas the TP53 mutation was exclusively detected in the post-treatment tissue of another individual.

Regarding the non-BRCA1/2 HR gene alterations detected by CGP, a RAD51C mutation was detected in a patient with a high GIS both before and after NACT. Additionally, a low-penetrance CHEK2 alteration was detected in a GIS-negative case.

TMB was high (>10 muts/MB) in 20% of pre-NACT tissues and in 10% of the post-NACT setting. Only in one patient were the pre- and post-treatment high-TMB results concordant.

### 3.6. Comparison of TCC, GIS, and TMB Pre- and Post-NACT 

Variations in TMB, TCC, and VAF were identified between paired tissues acquired prior to and following treatment.

**Figure 1:**
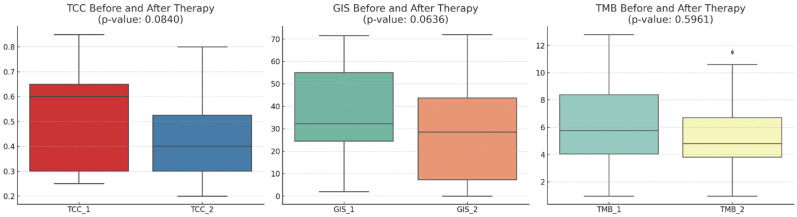
A comparison of the tumor cell content (TCC), genomic instability score (GIS), and tumor mutational burden (TMB) before and after therapy across 20 patients. The mean TCC decreased slightly after therapy, indicating a trend toward a reduction in the tumor cell count. The mean GIS score also showed a decrease after therapy, suggesting a trend towards genomic stability. The mean TMB score showed a slight decrease, but this change is not as pronounced as the others. With diamonds the outliers are visualized.

**Figure 2:**
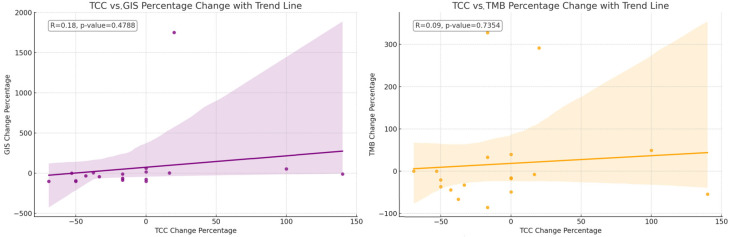
The correlation of the change in percentage for the value of TCC relative to the change in percentage for the value of GIS. The correlation of the change in percentage for the value of TCC relative to the change in percentage for the value of TMB. The relative changes in TMB and GIS before and after therapy do not correlate with the extent of the change in the TCC.

The mean TCC decreased slightly numerically after therapy (*p*-value: 0.0840), indicating a trend toward a reduction in cancer cells present in the samples obtained post-NACT. The mean GIS score also decreased numerically after therapy (*p*-value: 0.0636), suggesting a trend toward increased genomic stability. The mean TMB score slightly decreased (*p*-value: 0.5961), but this change was not as pronounced as the others (Figure 1). The relative changes in TMB and GIS before and after therapy did not correlate with the extent of the TCC change (Figure 2).

Comparing the TCC content before and after therapy, a more pronounced reduction was revealed in HRD-positive cases than in HRD-negative cases (*p*-value = 0.0641 vs. 0.4975), suggesting that the treatment may have been more effective in these cases due to the more significant reduction of the tumor.

**Figure 3:**
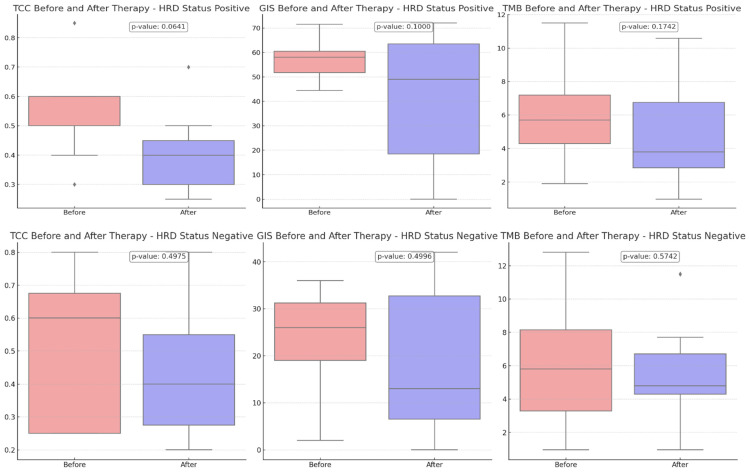
Comparison of tumor cell content (TCC), genomic instability score (GIS), and tumor mutational burden (TMB) before and after therapy, stratified by HRD status. Upper charts: HRD-positive patients (8 participants), Bottom Charts: HRD-negative patients (12 participants). With diamonds the outliers are visualized.

**Figure 4:**
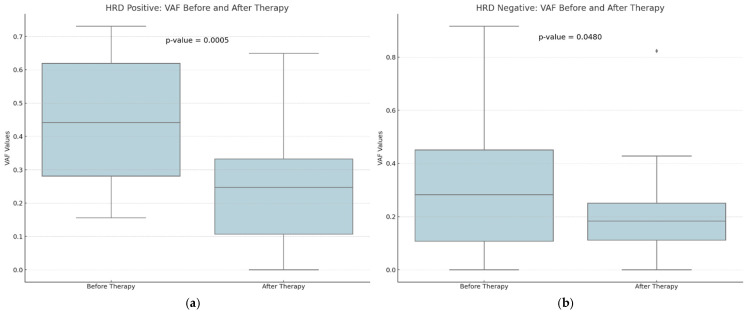
A comparison of the distribution of Variant Allele Frequency (VAF) values for HRD-positive (**a**) and HRD-negative (**b**) patients before and after therapy. There seems to be a higher decrease in the VAF values among HRD-positive patients after therapy. With diamonds the outliers are visualized.

A downward trend in GIS scores was observed between eight HRD-positive and twelve HRD-negative participants, although this trend did not reach statistical significance (*p*-value = 0.1000 versus 0.4996) (Figure 3). The VAF values detected by CGP exhibited the most pronounced difference, and they were substantially decreased in the post-treatment samples (*p*-value = 0.0015). Tumor samples obtained from HRD-positive patients before treatment exhibited a greater prevalence of VAF reduction than samples obtained from HRD-negative participants (*p*-value = 0.0005 vs. 0.0480) (Figure 4).

The mean TCC decreased after therapy among HRD-positive patients, indicating a trend toward a reduction in the tumor cell count.

### 3.7. VAF Changes

Moreover, the change in Variant Allele Frequency (VAF) and TCC before and after therapy were correlated. The correlation coefficient suggests a moderate positive correlation, and the *p*-value indicates that this correlation is statistically significant for GIS-positive and GIS-negative patients (r = 0.36, *p*-value = 0.00295).

### 3.8. CRS Correlations

**Figure 5:**
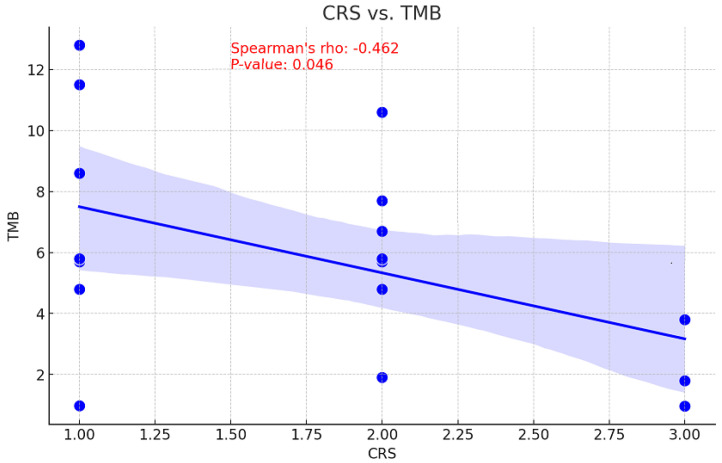
Correlation between CRS and TMB values.

**Figure 6:**
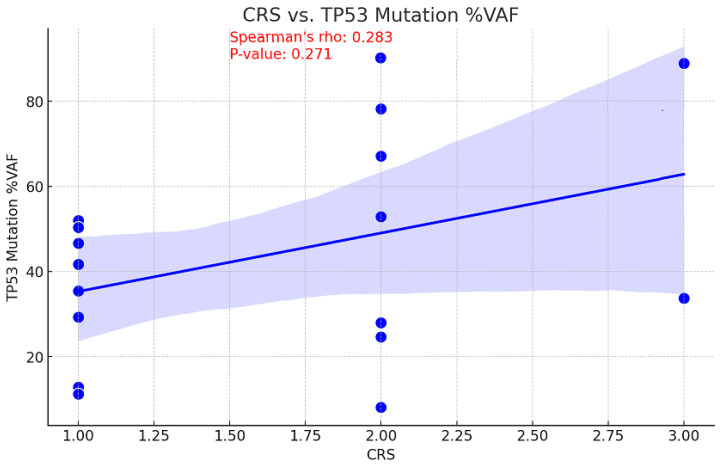
Correlation between CRS and TP53 mutation Variant Allele Frequency.

The relationship between tumor mutational burden (TMB) and chemotherapy response is unclear. While a high TMB may increase sensitivity to DNA-damaging treatments by decreasing cell viability, it may also suggest there are resistance mutations that diminish the efficacy of chemotherapy [17]. Consequently, the correlation between CRS and TMB values before NACT was examined to assess the ability of TMB to predict treatment outcomes. This information could be used to inform personalized therapy strategies and enhance patient management. With a *p*-value of 0.046, a moderate inverse relationship was observed between pre-NACT TMB and CRS, indicating this correlation is statistically significant (Figure 5). An association of moderate positivity (rho = 0.283, *p* = 0.271) was identified between CRS and TP53 gene VAF reduction; however, this relationship failed to achieve statistical significance (Figure 6).

A negative correlation between CRS and TCC change was indicated by the Spearman’s rank correlation coefficient test, which computed a correlation coefficient of 0.382. Based on the obtained *p*-value of 0.097, it can be concluded that this correlation lacks statistical significance at the 0.05 level.

## 4. Discussion

In advanced stages III and IV ovarian cancer, a series of studies have determined that the combination of NACT and intermediate cytoreduction is not inferior to initial cytoreduction and adjuvant chemotherapy. In addition, based on recent studies, maintenance therapy with PARP inhibitors, depending on the tumor molecular analysis (including BRCA1/2 mutations and HRD status), is recommended for all patients with advanced ovarian cancer. In this context, we aimed to determine the HRD status and tumor molecular profile and investigate potential differences in paired patients’ samples, both pre- and post-NACT, that could influence treatment decisions.

We identified that HRD positivity exhibited no significant change following NACT compared to pre-NACT tumor tissue, with a trend toward reduction (40% vs. 35%). This decline may also be ascribed to the reduced TCC content after the treatment. An elevation in the GIS status was noted in a single instance. This could be attributed to tumor heterogeneity or a more adequate sample collected after treatment, as the TCC for the post-NACT sample was 60%, whereas it was only 30% for the pre-NACT sample. This finding is consistent with prior research indicating that the HRD status remains consistent between the pre-and post-NACT samples of ovarian cancer patients, notwithstanding the genomic alterations that arise during recurrence [21].

Furthermore, upon analyzing the 20 patients for whom GIS results were accessible both prior to and after therapy, it was noted that a more pronounced reduction was observed in HRD-positive participants (*p*-value = 0.0641 vs. 0.4975), suggesting that the treatment may have been more effective in these instances where the reduction was more pronounced. Concerning the decrease in GIS scores, however, it failed to attain statistical significance.

The selection of the optimal specimen for analysis is critical in ensuring the accuracy of the outcomes acquired. Therefore, obtaining ascitic fluid to confirm malignancy is ineffective in the absence of an adequate quantity of cancer cells to enable a dependable molecular analysis. Insufficient TCC content was obtained for analysis in seven patients [22]. Low TCC was detected in the pre-NACT specimens of two patients and the post-NACT samples of five patients. It is noteworthy that four out of five of these patients had HRD-positive pre-NACT status, two of which had BRCA1 mutations, and the fifth had a GIS score approaching positivity (GIS 40). This may provide insight into the efficacy of NACT treatment for patients with HRD. Based on these findings, it is recommended that treatment-naïve samples undergo HRD testing. This is particularly important in cases where HRD is positive, as any decrease in tumor content can compromise the analysis.

This finding offers additional evidence favoring the hypothesis that the therapy successfully reduced VAF values. A negative correlation between CRS and TCC change has been observed in support of this. However, it did not reach statistical significance, possibly owing to the small number of tested patients.

Importantly, if preoperative chemotherapy is chosen, a biopsy containing a proportionate amount of cancer cells (greater than 30%) is imperative to conduct a molecular analysis of the tumor that yields solid outcomes.

Additionally, if intermediate cytoreduction follows effective preoperative chemotherapy, the residual disease may be minimal, as indicated by the extremely low percentage of remaining cancer cells and the unreliability of molecular analysis. Consequently, conducting a molecular analysis of the tumor obtained during the initial diagnosis excision becomes even more imperative. The impact of NACT on the molecular profile of ovarian cancer tumors is poorly understood, as evidenced by studies that analyzed a negligible number of samples or employed restricted gene panels. Nevertheless, it was observed that altered genes differ between tissues obtained before and after treatment, and this discrepancy may be associated with the response to the treatment [23,24,25]. Furthermore, recent research has demonstrated a decreased incidence of TCC in patients who have undergone NACT, suggesting that it is optimal to submit pre-chemotherapy biopsies [26]. The aforementioned observations suggest that NACT does indeed affect the molecular profile.

A molecular profile analysis of the tumors obtained from the 20 patients whose HRD results were accurate both before and after NACT revealed alterations in the detected gene, as well as in the frequency of the alterations detected. A moderate inverse relationship was observed between pre-NACT TMB and CRS. This finding contradicts previous research that suggested no differences in TMB between different CRS patient groups [27,28,29,30,31]. In addition, the reduction in the TCC content obtained after NACT was more pronounced in CRS3 patients, suggesting that responsive patients experience a greater degree of tumor reduction.

A decreased quantity of variations is identified in specimens after NACT as opposed to those before NACT (53 vs. 62). Additionally, a decrease in the VAF of the identified modifications was also observed and appears to be a correlation between the change in VAF and the change in TCC prior to and following therapy.

These results demonstrate that the proportion of cancer cells in both the initial biopsy and the intermediate cytoreduction sample significantly impacts the molecular analysis results, verifying the scant information currently available.

The observed decrease is assumed to result from the therapeutic effect; however, its potential as a prognostic indicator or predictor of the patient’s response to treatment has yet to be established.

There is no extensive bibliography related to the subject we are investigating. Two interesting papers deserve to be mentioned.

Arend et al. compared genetic variants by DNA next-generation sequencing [NGS] and gene expression in the tumor from 20 patients with HGSOC before and after NACT [24]. There was no significant difference in the number of variants seen in pre-and post-NACT, but of the 59 variants in the plasma pre-NACT, only 6 persisted, whereas 33 of 38 specific variants in the tumor DNA remained unchanged. Pathway analysis showed the most significant alterations in the cell cycle and DNA damage pathways.

Lodewijk et al. studied the genomic landscape and immune-related gene expression profiling of epithelial ovarian cancer after NACT [31]. They did not find major differences in the mutational landscape in paired biopsy and surgery samples, but a genomic loss of heterozygosity was found to be higher in patients with total/near-total tumor response. To our knowledge, this is the first paired analysis that examines the effect of NACT on HRD status and, more specifically, GIS as a biomarker of tumor response.

Our study’s limitations are the small number of paired tumor samples and the retrospective nature of data collection. Our understanding of molecular changes in correlation to NACT responsiveness was also compromised by the inability to obtain precise molecular analysis and GIS results from all post-NACT patients included in the study due to the TCC reduction. Any modifications in these areas would need to be taken into account when making therapeutic decisions following cytoreduction, and such considerations would need to be assessed at the initial therapeutic planning, especially in the era of PARP inhibitors.

## 5. Conclusions

In conclusion, the acquisition of adequate tumor tissue was shown to be of great importance to the outcome of molecular analysis of ovarian cancer when NACT is used. It is critical to obtain tumor tissue prior to NACT since clinical response may lead to insufficient residual tumor tissue or even necrotic material. In this case, valuable molecular data will be evaded. However, there are difficulties in performing tissue biopsies to detect ovarian cancer, such as the advanced stage and affected patient’s performance status. As a result, alternatives such as FNA or cytological examination of the ascitic fluid are usually preferred. It should be noted that noninvasive methods, like ctDNA sequencing at different time points, can be useful for customizing treatment. Our findings suggest the existence of tumor evolution or heterogeneity and indicate that new treatment options may become available, which may not be evident from the analysis of initial tumor biopsies.

Our results highlight the significant impact of NACT on the molecular characteristics of ovarian cancer, particularly in HRD-positive cases, and underscore the importance of considering these changes in therapeutic decision-making. Prospective studies with a large number of patients may shed more light on these clinically relevant challenges.

## Figures and Tables

**Table 1:** Patients’ clinical characteristics.

Characteristics	No. of Patients 20
Age at diagnosis (years) Median (range)	59.9 (39.78)
Initial FIGO stage	
Stage III	19
Stage IV	1
Histology	
High grade serous	20
Other	0
Chemotherapy response score	
CRS1	9
CRS2	8
CRS3	3
At initial diagnosis	
Cytology	1
Biopsy	19

## Data Availability

GIS and NGS analysis row data are available upon request to interested researchers.

## Supplementary Materials

The following supporting information can be downloaded at: https://www.mdpi.com/article/10.3390/cancers16213580/s1, Figure S1: Consort diagram; Table S1: Gene included in the panel used for tumor molecular profiling; Table S2: NGS analysis results in the 40 samples with evaluable HRD status.
